# Potential health impact and cost-effectiveness of bivalent human papillomavirus (HPV) vaccination in Afghanistan

**DOI:** 10.1016/j.vaccine.2019.12.013

**Published:** 2020-02-05

**Authors:** Palwasha Anwari, Frédéric Debellut, Elisabeth Vodicka, Andrew Clark, Farhad Farewar, Zubiada A. Zhwak, Dastagger Nazary, Clint Pecenka, D. Scott LaMontagne, Najibullah Safi

**Affiliations:** a Afghanistan National Immunization Technical Advisory Group, District 10, Kabul, Afghanistan; b PATH, Rue de Varembé 7, 1202 Geneva, Switzerland; c PATH, 2201 Westlake Ave, Suite 200, Seattle, WA 98121, USA; d London School of Hygiene and Tropical Medicine, Keppel Street, London WC1E 7HT, United Kingdom; e Health Economics and Financing Directorate, Ministry of Public Health, Masood Square, District 10, Kabul, Afghanistan; f Kabul University of Medical Sciences “Abu Ali Ibn Sina”, Jamal Mena, University Area, District 3, Kabul, Afghanistan; g Expanded Program on Immunization, Ministry of Public Health, Street 13, Wazir Akbar Khan, District 10, Kabul, Afghanistan; h World Health Organization, UNOCA Compound, Jalalabad Road, District 9, Kabul, Afghanistan

**Keywords:** Human papillomavirus, HPV vaccine, Cervical cancer, Cost-effectiveness analysis, Afghanistan

## Abstract

**Introduction:**

Human papillomavirus (HPV) vaccination has not been introduced in many countries in South-Central Asia, including Afghanistan, despite the sub-region having the highest incidence rate of cervical cancer in Asia. This study estimates the potential health impact and cost-effectiveness of HPV vaccination in Afghanistan to inform national decision-making.

**Method:**

An Excel-based static cohort model was used to estimate the lifetime costs and health outcomes of vaccinating a single cohort of 9-year-old girls in the year 2018 with the bivalent HPV vaccine, compared to no vaccination. We also explored a scenario with a catch-up campaign for girls aged 10–14 years. Input parameters were based on local sources, published literature, or assumptions when no data was available. The primary outcome measure was the discounted cost per disability-adjusted life-year (DALY) averted, evaluated from both government and societal perspectives.

**Results:**

Vaccinating a single cohort of 9-year-old girls against HPV in Afghanistan could avert 1718 cervical cancer cases, 125 hospitalizations, and 1612 deaths over the lifetime of the cohort. The incremental cost-effectiveness ratio was US$426 per DALY averted from the government perspective and US$400 per DALY averted from the societal perspective. The estimated annual cost of the HPV vaccination program (US$3,343,311) represents approximately 3.53% of the country′s total immunization budget for 2018 or 0.13% of total health expenditures.

**Conclusion:**

In Afghanistan, HPV vaccine introduction targeting a single cohort is potentially cost-effective (0.7 times the GDP per capita of $586) from both the government and societal perspective with additional health benefits generated by a catch-up campaign, depending on the government′s willingness to pay for the projected health outcomes.

## 1 Introduction

Cervical cancer is caused by the human papillomavirus (HPV). More than 100 HPV types have been identified, about 40 of which can infect the genital area. Two high-risk types of HPV, 16 and 18, account for about 70% of all cervical cancer cases. Cervical cancer is the second most common cancer in women living in lower-income regions with an estimated 570,000 new cases and 311,000 deaths annually [1], [2]. While pre-cancerous cervical lesions and cervical cancer are treatable if detected early, 85% of these deaths occur in low- and middle-income countries (LMICs) where routine cervical cancer screening and treatment are not widely available. Across Asia, age-standardized incidence rates vary from 4.4 to 19.3 per 100,000 person-years with South-Central Asia having the highest incidence rate (19.3 per 100,00 person-years) of cervical cancer across Asia [3], [4]. In Afghanistan, for example, current estimates indicate that 862 women are diagnosed with cervical cancer every year and 570 die from the disease [5]. Afghanistan does not currently have a national screening program for cervical cancer [6], making primary prevention through vaccination particularly important.

Since 2009, the World Health Organization (WHO) has recommended vaccination as a primary prevention measure against cervical cancer with a suggested target population of 9- to 14-year-old girls [7]. Nonetheless, the vaccine has not yet been introduced in many South-Central Asian countries, including Afghanistan. There are currently three WHO-prequalified HPV vaccines available on the market: a bivalent vaccine (Cervarix®, produced by GlaxoSmithKline) that protects against HPV types 16 and 18; a quadrivalent vaccine (GARDASIL®/Silgard®, produced by Merck & Co.) that protects against HPV types 16 and 18 as well as types 6 and 11, which are responsible for anogenital warts; and a nonavalent vaccine (Gardasil 9/Merck & Co.) that protects against types 6, 11, 16, 18, and the additional oncogenic types 31, 33, 45, 52, and 58 [8]. As of mid-2019, HPV vaccines have been introduced in 93 countries using a variety of delivery strategies including school-based, health facility, and community-based programs [9].

Several studies suggest that HPV vaccination would be a cost-effective intervention for the prevention of cervical cancer in most countries [10], [11]. However, to our knowledge, no data exists on the potential health, financial, or economic impact of introducing the vaccine in Afghanistan. Further, cost-effectiveness analyses of vaccination in neighboring countries, such as Iran, India, China and other countries in Central Asia, have provided mixed results [12], [13], [14], [15]. Individual country-level characteristics, local vaccine pricing, and costs need to be considered to sufficiently inform governments on the potential value of HPV vaccination.

Afghanistan benefits from financial support from Gavi, the Vaccine Alliance for introduction of new vaccines in the national immunization program. There is an increasing need for the Afghan government to develop a strong and robust evidence base for public health priority-setting. Since 2002, Afghanistan primary healthcare services have been almost totally dependent on donor funding to support the rebuilding of the health system following the collapse of the Taliban regime. However, in recent years, the country has experienced a drastic decrease in donor funding. In part to address subsequently tighter budgets, the Ministry of Public Health (MoPH) is in the process of critically appraising currently funded health interventions and assessing the cost-effectiveness of existing and future investments. Following that exercise, MoPH will revise national healthcare investments to more efficiently meet the health needs of the population under constrained resources.

In 2016, in-country experts on immunization and rotavirus disease undertook a study evaluating the health impact and cost-effectiveness of rotavirus vaccination in Afghanistan. Study results suggested good value for money with an incremental cost-effectiveness ratio (ICER) of US$82 per disability-adjusted life-year (DALY) averted. The projected average yearly cost of a rotavirus vaccination program represented 2.8% of total immunization costs expected in 2017 or 0.1% of total health expenditures [16]. Subsequently, the country introduced rotavirus vaccine in January 2018. To support the MoPH in identifying other high-value vaccines for Afghan women and families, we conducted this study to examine the potential health impact and cost-effectiveness of introducing the HPV vaccine.

## 2 Methods

We compared the potential costs and health consequences of HPV vaccination with no vaccination at the national level. A multidisciplinary team of experts from the National Immunization Advisory Group (NITAG); the Health Economics and Financing Directorate (HEFD); the Reproductive, Maternal, Neonatal, Child and Adolescent Health Directorate (RMNCAH); the National Expanded Program on Immunization (NEPI); the National Cancer Control Program (NCCP); and the World Health Organization (WHO) country office in Afghanistan carried out this study with support of PATH and London School of Hygiene and Tropical Medicine (LSHTM). Considering the country context, vaccine price, delivery costs, and licensed indications, the research team elected to explore introduction of the bivalent vaccine, Cervarix® (GlaxoSmithKline Biologicals S.A.). The bivalent vaccine is a 2-dose presentation [17] to be administered with at least six months between the first and second dose.

In the analysis, our base-case scenario assumes a vaccination target population of one cohort of girls aged 9 years old in the year 2018, relying on demographic projections (population size and life expectancy by age, sex, and calendar year) over the lifetime of the 2009 birth cohort in Afghanistan [18]. We also assessed the marginal costs and benefits of including a one-time national catch-up campaign of 10- to 14-year-old girls. The base-case scenario reflects the lifetime costs and benefits of an ongoing vaccination program, while the catch-up campaign scenario examines the additional benefits and costs of vaccinating five additional cohorts (10–14 years old) in the initial year. In short, the catch-up campaign examines five additional cohorts (10–14 years old) in addition to the 9-year-old cohort.

This economic evaluation examines results from the government perspective (vaccine program costs only) and the societal perspective (vaccine program costs plus direct and indirect costs of cervical cancer treatment) over the lifetime of the cohorts under consideration. The primary outcome of the analysis is the cost per disability-adjusted life-year (DALY) averted through vaccination. Other outcomes include the number of cases, hospitalizations, deaths, and treatment costs with and without vaccination, as well as the incremental cost of the vaccination program.

## 3 Model

We used the UNIVAC decision-support model (version 1.4) for the analysis. UNIVAC is an established static cohort model developed in Excel (Excel, Microsoft Corp, Redmond, WA, US) [19]. It was specifically designed for use in LMICs by national health ministries and partners. It aims to produce conservative estimates of impact and cost-effectiveness of new vaccine introduction to inform national vaccine policy decisions. The model can be customized to reflect the disease categories and age group(s) of interest. Model input parameters include demographic data of the target population, cervical cancer incidence and mortality by stage (defined in the model as local, regional and distant), vaccine coverage and efficacy, costs of the vaccination program, and cervical cancer treatment costs. The model is a simplified representation of natural history of disease and does not incorporate progression to or regression from different grades of cervical intraepithelial neoplasia. Additionally, we assumed that no women receive cervical cancer screening to reflect the current practice in the country. Data to inform inputs were gathered from the published literature, published/unpublished local sources and local experts′ consultations, or based on assumptions when no information was available. All model inputs were discussed by a multidisciplinary team of experts led by an in-country lead researcher and a health economist from PATH. The expert team was consulted frequently between April and October 2018 to achieve consensus on relevant scenarios, identify appropriate data sources, and finalize input parameters. All monetary terms were adjusted to 2018 US$ using currency exchange rates from the central bank of Afghanistan [20] (exchange rate at time of analysis: US$1 equals 73.5 Afghani (AFN)) and the U.S. Consumer Price Index [21]. We applied a 3% discount rate for future costs and health outcomes in our calculations [22].

## 4 Disease burden

We used the United Nations Population Division (2017 revision) database to estimate the size of the target population over time, assuming a lifespan of 100 years [18]. Cervical cancer incidence was estimated by 5-year age groups and stage of cancer (local, regional, and distant). We used the Federation of International Gynecology and Obstetrics (FIGO) classification for cervical cancer to define local (stage IA, IIA, and IB1), regional (IB2, IIB, and IIIB), and distant (IVB and IVA) stages [23]. Due to the absence of a national cancer registry and local epidemiology studies, we used international estimates to project the burden of cervical cancer in Afghanistan based on GLOBOCAN, the global database from the International Agency for Research on Cancer (IARC). GLOBOCAN provides estimates of the incidence and mortality of major cancer types by age group for 184 countries [21]. For Afghanistan, GLOBOCAN estimates an age-standardized rate of cervical cancer incidence of 8.8 per 100,000 women per year and a mortality rate of 6.9 per 100,000. Age-specific GLOBOCAN rates used in our analysis are shown in Table 1. We assumed there would be no change in cancer incidence and mortality rates over time. For DALY calculations, we applied disability weights for cervical cancer of 0.288, 0.451, and 0.540 for local, regional, and distant cases, respectively, based on the most recent Global Burden of Disease study data [24]. We also assumed an average duration of illness (i.e., time spent living with disease) of 10, 7.5, and 2 years for local, regional, and distant cervical cancer, respectively, based on 5-year survival rates from India and validated by in-country oncology experts [25].

**Table 1:** Input parameters for estimating cervical cancer disease burden.

Parameters	Estimate	Source (s)		
Age- Specific rates 100,000 per year, local cervical cancer cases				
10–14 years old	0.00	[5]		
15–19 years old	0.86			
20–24 years old	0.86			
25–29 years old	0.86			
30–34 years old	0.86			
35–39 years old	0.86			
45–49 years old	5.02			
50–54 years old	5.44			
55–59 years old	0.09			
60–64 years old	3.80			
65–69 years old	2.85			
70–74 years old	2.16			
75–79 years old	1.46			
80–84 years old	1.46			
85–89 years old	1.46			
90–94 years old	1.46			
95–99 years old	1.46			
*Age-specific rates per 100,000 per year, regional cervical cancer cases*				
10–14 years old	0.00	[5]		
15–19 years old	3.36			
20–24 years old	3.36			
25–29 years old	3.36			
30–34 years old	3.36			
35–39 years old	3.36			
40–44 years old	15.94			
45–49 years old	19.68			
50–54 years old	21.32			
55–59 years old	19.51			
60–64 years old	14.88			
65–69 years old	11.15			
70–74 years old	8.46			
75–79 years old	5.74			
80–84 years old	5.74			
85–89 years old	5.74			
90–94 years old	5.74			
95–99 years old	5.74			
*Age-specific rates per 100,000 per year, distant cervical cancer cases*				
10–14 years old	0.00	[5]		
15–19 years old	0.39			
20–24 years old	0.39			
25–29 years old	0.39			
30–34 years old	0.39			
35–39 years old	0.39			
40–44 years old	1.86			
45–49 years old	2.30			
50–54 years old	2.49			
55–59 years old	2.28			
60–64 years old	1.74			
65–69 years old	1.30			
70–74 years old	0.99			
75–79 years old	0.67			
80–84 years old	0.67			
85–89 years old	0.67			
90–94 years old	0.67			
95–99 years old	0.67			
*Age-specific rates per 100,000 per year, cervical cancer deaths*				
10–14 years old	0.00	[5]		
15–19 years old	1.51			
20–24 years old	1.51			
25–29 years old	1.51			
30–34 years old	1.51			
35–39 years old	1.51			
40–44 years old	11.41			
45–49 years old	17.93			
50–54 years old	23.30			
55–59 years old	25.49			
60–64 years old	24.91			
65–69 years old	23.23			
70–74 years old	19.64			
75–79 years old	14.76			
80–84 years old	14.76			
85–89 years old	14.76			
90–94 years old	14.76			
95–99 years old	14.76			
*Disability weights for DALY calculation*				
Disability weight (local)	0.288	[24]		
Disability weight (regional)	0.451			
Disability weight (distant)	0.540			
*Average number of years living with cervical cancer*				
	*Estimate*	*Low*	*High*	*Source (s)*

Local	10.00	7.50	15.00	Assumption based on expert consultation
Regional	7.50	5.00	10.00	
Distant	2.00	1.00	3.00	

## 5 Vaccine efficacy and coverage

The impact (% reduction) in cervical cancer cases and deaths following vaccine introduction was estimated to be 45%. This was calculated by multiplying together (i) vaccine coverage, assumed to be 70% of the target cohort (i.e. 9-year-old girls and (ii) vaccine effectiveness (VE), assumed to be 65% after two doses. VE was estimated by multiplying vaccine-type coverage, assumed to be 69% based on the proportion of cervical cancer caused by types 16 and 18 in the Eastern Mediterranean region [26] by the efficacy against types 16 and 18, assumed to be 94%. This is the mean of the efficacy values reported in two pivotal trials (PATRICIA [27] and FUTURE II HPV [28]. The efficacy of one-dose vaccination is still uncertain; therefore, we made a conservative base case-assumption that one dose of the vaccine (i.e., an incomplete course) would confer half of the two-dose VE. Recent evidence suggests that a single dose may confer similar VE as two doses so we also ran a single-dose strategy with this assumption [29], [30]. In addition, we ran a scenario with the VE inflated to 40% for first dose and 80% for the second dose to account for potential cross-protection against non-vaccine types [31]. This scenario implies 48% efficacy after the second dose for the remaining 31% of cervical cancer caused by types other than 16 and 18 in the Eastern Mediterranean region [26]. Following vaccination, we assume life-long protection [32].

Afghanistan, like many other low-resource countries, has used mixed delivery strategies in national immunization programs to achieve high vaccination coverage. Available delivery strategies include fixed sites (e.g., health center), outreach (e.g., households in the catchment area of a health facility), mobile (e.g., mobile teams travel to remote and hard-to-reach geographical areas), and campaigns (e.g., school and household). To reach girls 9 to 14 years of age, school-based delivery will likely be the primary approach. We assumed 55% of the target population is in school according to the school attendance rate for Afghan girls at the age of 11 [33]. Forty-five percent of girls in the target population will have to be reached differently, such as via health facilities or community outreach activities. In consultation with the Expanded Program on Immunization and other key stakeholders, we assumed coverage rates of 70% and 65% for the first and second dose, respectively (Table 2). For the catch-up campaign scenario, we assumed an 80% and 75% coverage rate among girls aged 10–14 years for the first and second dose, respectively. Since some of the girls in a catch-up campaign will be beyond the age of sexual debut, we assumed the VE would be lower than the value assumed for 9-year-old girls (55% vs 65%).

**Table 2:** Input parameters for estimating the health impact of HPV vaccination.

Parameter	Estimate	Scenarios		Source (s)
		Low	High	
*Coverage in year of introduction*				
Dose 1	70%	55%	95%	Assumption based on expert consultation
Dose 2	65%	50%	90%	
*Coverage in first year campaign*				
Dose 1	80%	55%	95%	Assumption based on expert consultation
Dose 2	75%	50%	90%	
*Vaccine efficacy adjusted for vaccine types*				
After primary dose 1	32.50%	–	65%	[27], [28]
After primary dose 2	65%	–	–	
*Vaccine cross-protection efficacy*				
After primary dose 1	40%			[32]
After primary dose 2	80%			

## 6 Health service utilization

Cervical cancer treatment options in Afghanistan are limited. Only one public health facility–Jamhuryat Hospital in Kabul–provides more advanced treatment options. Jamhuryat Hospital has a 60-bed tertiary care unit that provides basic diagnostic tests, surgeries, and administration of chemotherapy medicines to a limited number of patients who are required to purchase their own medicines outside of the hospital. Patients who can afford to seek care typically do so in the private sector where they bear the entire cost of treatment. Radiotherapy, one of the most effective treatments for local and regional cervical cancer, is not available anywhere in the country. We assumed 15% of women referred to radiotherapy would travel to India and Pakistan to seek these services. In the absence of a screening program, cancer would only be detected when women seek care for symptoms. We assumed that 1.74%, 7.35%, and 17.46% of local, regional, and distant cervical cancer cases, respectively, are detected symptomatically [34]. Annex 1 in supplementary file illustrates the typical set of activities required to seek treatment for cervical cancer in Afghanistan.

## 7 Healthcare costs

We estimated the direct medical, non-medical, and indirect costs of cervical cancer diagnosis and treatment in private health facilities. To estimate costs in the absence of data, we sought the expert opinion of in-country clinicians and MoPH and NCCP officials. We used an ingredients-based method to estimate costs separately for local, regional, and distant cervical cancer. Direct medical costs were based on established hospital fees for services. We estimated non-medical costs accounting for accommodation, food, and transportation costs incurred while seeking care. We also calculated the indirect opportunity costs of seeking care for both the female patient and her attendee. For each treatment strategy, we estimated the number of days the pair would need to miss from work or usual activities (e.g., taking care of children, leisure activities) due to diagnosis and treatment. We calculated costs associated with loss of productivity based on Afghanistan′s per capita Gross Domestic Product (GDP), accounting for the number of days lost. Input values are displayed in Table 3 and detailed calculations for healthcare costs are available in Annex 2 in supplementary file.

**Table 3:** Input parameters for estimating health service utilization, health service cost and vaccine program cost (all costs are presented in 2018 US$).

Parameter	Estimate	Scenarios		Source (s)
		Low	High	
*Treatment seeking proportion (identified via symptoms)*				
Local	1.74%	0.87%	3.48%	[34]
Regional	7.35%	3.675%	14.7%	
Distant	17.46%	8.73%	34.92%	
*Health service cost*				
*Household cost per treated woman*				
Local cancer	$4,816.18	$3,315.71	$6,273.11	Cost calculations see Annex 1
Regional cancer	$5,715.62	$4,215.15	$8,060.68	
Distant cancer	$5,132.13	$5,132.13	$6,616.32	
*Vaccine program cost*				
Vaccine price per dose (routine)	$0.20	–	$4.60	[17], [35], [36]
Vaccine price per dose (campaign)	$0.0	–	$4.60	[37]
Percentage of international handling	3.0%	–	–	[41]
Percentage of international delivery	4.0%	2.0%	6.0%	
Percentage of wastage	10.0%	–	–	[40]
Cost of syringes (price per dose)	$0.05	–	-	[42]
Safety box (price per dose)	$0.01	–	–	
Incremental health system cost (routine)	$4.59	$1.73	$6.32	[38], [46]
Incremental health system cost (campaign)	$1.73	–	–	

## 8 Vaccination program costs

The last known vaccine price secured by Gavi for Cervarix is US$4.60 per dose [17]. Afghanistan is eligible for Gavi support, falling in the initial self-financing transition phase. In this transition phase, country co-financing is US$0.20 per dose for routinely administered vaccines [35], [36]. Countries remain in this transition phase as long as their per-capita Gross National Income (GNI) remains below US$1045 [35]. For vaccines administered as a catch-up campaign, Gavi would support HPV vaccines′ procurement entirely [37]. In our base-case scenario, we use the country co-financing rate and explore the impact of the country graduating to pay the full vaccine price in an alternative scenario.

Due to the unavailability of country-level estimates for HPV vaccination delivery, we used an average economic cost of US$4.59 per dose based on the Immunization Costing Action Network (ICAN) data reported by studies from five LMICs covering a mix of vaccine delivery strategies [38]. In alternative scenarios, we applied a lower delivery cost of US$1.73 per dose, as used previously by Campos et al. to model HPV vaccine impact in 50 LMICs [39]. For the catch-up campaign we applied the lower delivery cost of US$1.73 per dose, considering the campaign will reach a larger group per vaccination session leading to a lower cost per dose delivered.

We applied a wastage rate of 10% [40] and applied 3% and 4% of the vaccine price to account for international handling and delivery, respectively [41]. We account for US$0.05 and US$0.01 per dose for syringes and safety boxes, respectively [42] (Table 3).

## 9 Sensitivity analyses

We conducted univariate sensitivity analyses to identify the impact of uncertainty introduced by individual study parameters on model outcomes. We conducted additional sensitivity analyses to model variations in vaccine price, health service utilization, healthcare treatment costs, incremental health system costs, vaccine coverage, and vaccine efficacy.

## 10 Results

### 10.1 Base-case scenario

The UNIVAC model estimated that, without vaccination, 4026 cervical cancer cases, 281 hospitalizations, 3678 deaths, and 17,919 DALYs would occur in the lifetime of a single cohort of girls aged 9 years old. Introducing HPV vaccine targeting a single cohort of 9-year-olds without a catch-up campaign is projected to avert 1765 cervical cancer cases, 123 hospitalizations, 1612 deaths, and 7855 DALYs for a single year of vaccination. When the model was extended to include a catch-up campaign for additional cohorts of girls 10–14 years old, we projected that, in the absence of a vaccination program, there would be 19,569 cases, 1365 hospitalizations, 17,865 deaths, and 92,062 DALYs due to cervical cancer. In the 9- to 14-year-old targeted population, HPV vaccination would subsequently avert 9849 cases, 687 hospitalizations, 8991 deaths, and 46,332 DALYs. Table 4 provides the total number of cases, hospitalizations, deaths, and DALYs projected to occur with and without HPV vaccination under each scenario.

**Table 4:** Lifetime health outcomes of HPV vaccination of a single cohort of 9-year-old girls with and without an initial catch-up campaign among 10- to 14-year-old girls.

Single cohort of 9-year-old girls without catch-up campaign			
	No vaccine	With vaccine	Averted
**Total cervical cancer cases**	**4,026**	**2,261**	**1,765**
Local cancer	749	420	328
Regional cancer	2,934	1,648	1,286
Distant cancer	343	193	150
*Total cervical cancer Hospital visits*	*281*	*158*	*123*
Local cancer	14	8	6
Regional cancer	216	121	95
Distant cancer	51	29	23
*Deaths*	*3,678*	*2,066*	*1,612*
*DALYS (Discounted)*	*17,919*	*10,065*	*7,855*
*Single cohort of 9-year-old girls with catch-up campaign in additional five cohorts (10- to 14-year-old girls)*			
	*No vaccine*	*With vaccine*	*Averted*
*Total cervical cancer cases*	*19,569*	*9721*	*9849*
Local cancer	3639	1807	1831
Regional cancer	14,264	7085	7178
Distant cancer	1,667	828	839
*Total cervical cancer Hospital visits*	*1365*	*678*	*687*
Local cancer	67	34	34
Regional cancer	1048	521	528
Distant cancer	249	124	125
*Deaths*	*17,865*	*8874*	*8991*
*DALYS (Discounted)*	*92,062*	*45,730*	*46,332*

We estimate it would cost the government US$3,343,311 per year (3.53% of country′s total immunization budget for 2018) to vaccinate a single cohort of 9-year-old girls without a catch-up campaign and US$9,249,429 (9.76% of country′s total immunization budget for 2018) to vaccinate a cohort of 9-year-old girls with a catch-up campaign in the additional cohorts. HPV vaccination would prevent US$203,226 healthcare costs related to downstream cervical cancer treatment for one cohort of girls immunized or US$1,202,566 with the addition of a catch-up campaign in the additional cohorts, 10–14 years old (Table 5).

**Table 5:** Discounted lifetime costs of HPV vaccination of single cohort of girls 9-year-old girls with and without an initial catch-up campaign among 10- to 14-year-old girls.

Single cohort of 9-year-old girls without catch-up campaign			
	No vaccine	HPV vaccine	Delta
Total government healthcare cost	–	–	–
Total societal healthcare costs	463,633	260,407	203,226
Total vaccine program cost (government perspective)	–	3,343,311	–
*Single cohort of 9-year-old girls with catch-up campaign in additional five cohorts (10- to 14-year-old girls)*			
Total government healthcare cost	–	–	–
Total societal healthcare costs	2,391,556	1,188,989	1,202,566
Total vaccine program cost (government perspective)	–	9,249,429	–

Overall, we estimated that from the government perspective, HPV vaccination in Afghanistan would yield an ICER of US$426 (72.7% of GDP per capita) and US$200 (34.13% of GDP per capita) per DALY averted for a single cohort without and with a catch-up campaign, respectively. From the societal perspective, the ICER suggests that HPV vaccination would cost US$400 (68.3% of GDP per capita) and US$174 (29.7% of GDP per capita) per DALY averted for a single cohort without and with a catch-up campaign, respectively.

## 11 Sensitivity analysis

**Fig. 1:**
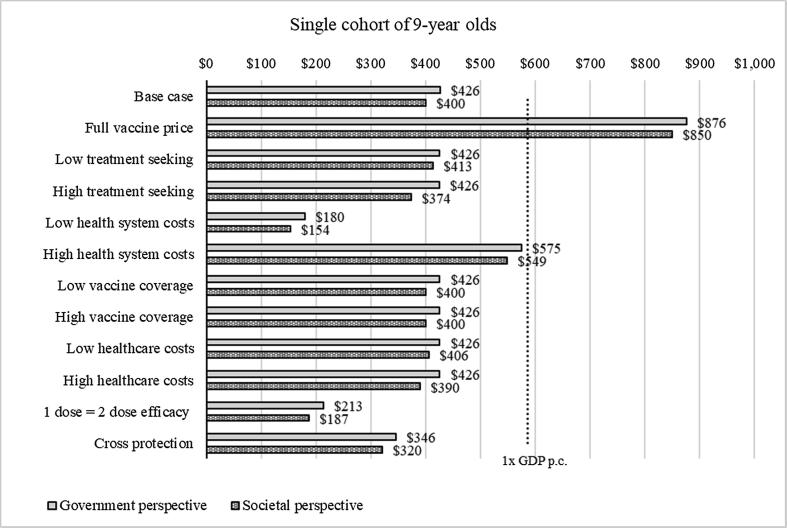
Sensitivity analysis for single cohort of girls aged 9 years old, 2018^a a^The figure show the incremental cost-effectiveness ratio for each scenario evaluated. The light gray bars show ICER from the government perspective, the dark grey bars show ICER from the societal perspective. The dotted line shows the cost-effectiveness threshold of one times GDP per capita.

**Fig. 2:**
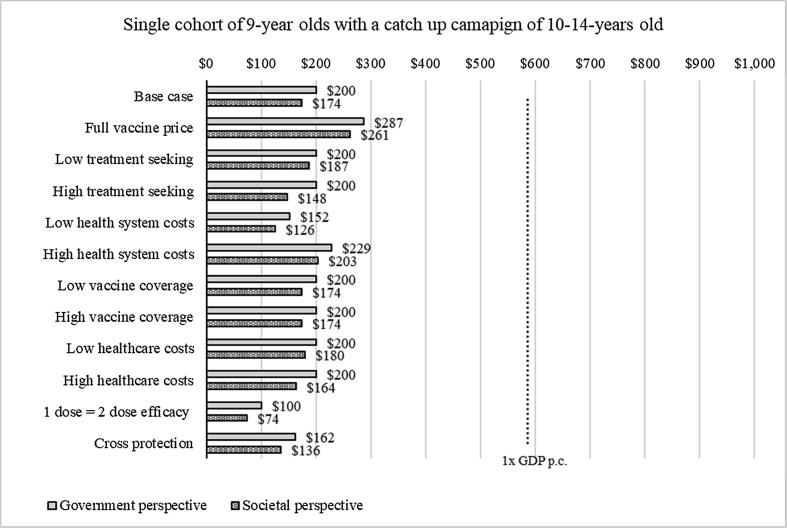
Sensitivity analysis for single cohort of girls aged 9 years old with catch-up campaign of 10- to 14-year-old girls, 2018^a^**^.^**^a^ The figure show the incremental cost-effectiveness ratio for each scenario evaluated. The light gray bars show ICER from the government perspective, the dark grey bars show ICER from the societal perspective. The dotted line shows the cost-effectiveness threshold of one times GDP per capita.

Results of the sensitivity analysis for HPV vaccination of 9-year-old girls showed that the estimated ICER ranged from US$180 to US$876 (0.3 to 1.5 times Afghanistan′s GDP per capita) from the government perspective. Table 6 details the values associated with parameter value changes. Scenarios applying high health system costs with co-financing and the full price of the vaccine resulted in higher ICERs of US$575 and US$867 (1–1.5 times GDP), respectively. From the societal perspective, the estimated ICER ranged from US$154 to US$850 (0.26 to 1.45 times GDP per capita) (Fig. 1). Similar uncertainty analyses were run for vaccination of 9-year-old girls with a catch-up campaign of 10- to 14-year-old girls. Results were similar with a cost per DALY averted ranging from US$152 to US$287 from the government perspective (0.25 to 0.49 times GDP per capita) and from US$126 to US$261 (0.22–0.45 times GDP per capita) from the societal perspective. The lowest ICERs were found when applying low incremental health system costs (Fig. 2).

**Table 6:** Parameters and values utilized in sensitivity analyses.

Scenario	Parameter	Base value	Sensitivity value	Source
Full vaccine price	Vaccine price per dose	$0.20	$4.60	[17]
Low treatment seeking	Treatment seeking proportion			[34]
	Local	1.74%	0.87%	
	Regional	7.35%	3.675%	
	Distant	17.46%	8.73%	
High treatment seeking	Treatment seeking proportion			[34]
	Local	1.74%	3.48%	
	Regional	7.35%	14.7%	
	Distant	17.46%	34.92%	
Low health system costs	Incremental health system cost (routine)	$4.59	$1.73	[38], [46]
High health system costs	Incremental health system cost (routine)	$4.59	$6.32	[38], [46]
Low vaccine coverage	Coverage in year of introduction			Assumption based on expert consultation
	Dose 1	70%	55%	
	Dose 2	65%	50%	
	Coverage in first year campaign			
	Dose 1	80%	55%	
	Dose 2	75%	50%	
High vaccine coverage	Coverage in year of introduction			
	Dose 1	70%	95%	
	Dose 2	65%	90%	
	Coverage in first year campaign			
	Dose 1	80%	95%	
	Dose 2	75%	90%	
Low healthcare costs	Household cost per treated woman			Cost calculations see Annex 1
	Local cancer	$4,816.18	$3,315.71	
	Regional cancer	$5,715.62	$4,215.15	
	Distant cancer	$5,132.13	$5,132.13	
High healthcare costs	Household cost per treated woman			Cost calculations see Annex 1
	Local cancer	$4,816.18	$6,273.11	
	Regional cancer	$5,715.62	$8,060.68	
	Distant cancer	$5,132.13	$6,616.32	
1 dose = 2 dose efficacy	Efficacy after primary dose 1	32.5%	65%	[27], [28]
Cross protection	Efficacy after primary dose 2	65%	80%	[32]

We also examined scenarios for higher vaccine efficacy, accounting for cross-protection against other types of HPV in addition to types 16 and 18. Results showed that ICERs decreased to US$346 and US$320 from the government and societal perspectives, respectively, in a single cohort of adolescent girls. When we examined cross-protection among 9-year-old girls with a catch-up campaign of five additional cohorts of girls, the cost per DALY averted decreased further to US$162 and US$136 from the government and societal perspectives. Finally, when we assumed a single dose of HPV vaccine would yield equivalent efficacy to a two-dose HPV vaccine schedule, the ICER decreased to one-third and one-fourth of the GDP per capita for a single cohort and a single cohort with a catch-up campaign with costs per DALY averted of US$146 and US$100, respectively. Results from the univariate sensitivity analyses indicate that introduction of HPV vaccination would likely be cost-effective from both the government and societal perspectives with additional benefits of adding a catch-up campaign of 10- to 14-year-old girls. Main model drivers include the vaccine price and the incremental health system costs.

## 12 Discussion

This analysis suggests that in Afghanistan, introduction of the bivalent HPV vaccine is likely cost-effective compared to no vaccination based on an established local willingness-to-pay threshold of one times the GDP per capita of US$586. ICERs ranged from US$426 (0.73 times GDP per capita) to US$200 (0.34 times GDP per capita) without and with a catch-up campaign, respectively, from the government perspective and US$400 and US$174 (0.68 and 0.30 times GDP per capita), respectively, from the societal perspective. The annual cost to implement an HPV vaccination program in routine vaccination only or with a catch-up campaign would represent approximately 3.53% or 9.76% of the country′s total immunization budget for 2018, or 0.13% and 0.35% of total health expenditures, respectively.

Drivers of the results are mainly the vaccine price and the incremental health system cost. This finding confirms results from other cost-effectiveness studies in the region, which suggest that a low vaccine price and cost per vaccinated girl are critical to achieving good value for money. For example, a cost-effectiveness analysis in Iran, an upper middle income country where the expected vaccine price is higher at approximately US$13–16, the vaccine was not found to be cost-effective when compared to a threshold of GDP per capita (US$14,289 in 2015) [12]. Contrasting results from India, a lower-middle income country with Gavi pricing, projected the vaccine to be very cost effective with an incremental cost per quality-adjusted life year gained of INR 73 or US$1.12 compared to a willingness to pay threshold of INR 10,000 or US$135 in 2016. An evaluation of Eastern European and Central Asian countries, including Afghanistan′s neighboring countries of Turkmenistan, Uzbekistan, and Tajikistan, found that cost per vaccinated girl was the key driver of cost-effectiveness [15]. While the cost of vaccine procurement is set by Gavi′s co-financing policy for qualifying countries, there is an incentive to minimize the cost to deliver the vaccine as much as possible by finding the correct strategy to reach girls. Further, for long-term sustainability of an HPV vaccination program, vaccine price setting needs to be achievable for countries that have graduated from Gavi eligibility but still may not be able to afford higher prices provided to countries that no longer qualify for UNICEF pricing. Single-dose vaccination may be a more attractive scenario economically, but more research on the long-term efficacy is required prior to adoption of such a strategy. HPV vaccination could have a profound positive impact on the long-term health outcomes of girls and women in Afghanistan, particularly given that cervical cancer screening is currently not available in the country and treatment services are limited. Additionally, families bear all healthcare costs out of pocket, making access to cervical cancer care out of reach for many women and their families. These health systems challenges are compounded by rapid demographic and epidemiologic transitions that have led South and Central Asian countries, including Afghanistan, to experience an increasing health burden of non-communicable diseases [43]. IARC estimated there were 20,000 cases of any type of cancer in Afghanistan in 2012. By 2030, this figure is expected to rise by 61% (almost 33,000) [44]. Cervical cancer is unique in that it is preventable, detectable, and treatable if addressed effectively. Tools for doing so have widely been accepted as cost-effective; however, incidence rates in many countries, including Afghanistan, continue to be unacceptably high, prompting WHO to call for global elimination of cervical cancer [45]. To support countries in addressing this call to action, evidence is needed to identify optimal and affordable strategies for a national approach. This study provides evidence regarding the potential health and economic impact of initiating HPV vaccination to reduce the burden of cervical cancer in Afghanistan.

The decision to introduce newer vaccines, such as rotavirus, pneumococcal, and HPV vaccines, requires careful attention toward affordability and cost-effectiveness due to their higher costs compared to earlier antigens. In 2017, Afghanistan conducted a cost-effectiveness analysis using the UNIVAC model exploring rotavirus vaccine introduction strategies [16]. Findings of that economic evaluation indicated that introduction of rotavirus vaccine compared to no vaccine was likely to be highly cost-effective based on a willingness-to-pay threshold of one times the GDP per capita (US$562). That study helped policymakers in Afghanistan make an informed decision on whether to include rotavirus vaccine in the national immunization program, which was operationalized in January 2018. Building off this success, we applied the same methodological strategy to evaluate the cost-effectiveness of HPV vaccination to provide similar decision support to the Government of Afghanistan. Similar to rotavirus vaccine, the potential budget impact of introducing HPV vaccination appears substantial despite financial support available from Gavi, while achievable health gains are projected to be lower. Vaccination program investments will need to be weighed against other health investment priorities for the MoPH.

This study has a number of limitations. First, we were not able to include the potential health and costs associated with other preventive interventions, such as screening or treatment for precancerous lesions. However, in an evaluation of the health and economic impact of scaling cervical cancer prevention in 50 low- and lower-middle-income countries, including Afghanistan, Campos et. al. found that a combined strategy of HPV vaccination of young girls with a cervical cancer screen-and-treat program could avert substantial burden of disease while providing good value for public health dollars [46]. Second, burden of disease data were not available for Afghanistan. Rather, we used GLOBOCAN estimates for age-specific incidence of cervical cancer based on a mean incidence in Afghanistan′s neighboring countries, Pakistan and Tajikistan, which could underestimate or overestimate the real burden of disease [5]. A nationwide comprehensive cancer registry would support future cancer-related decision-making in Afghanistan. Third, there were no data on healthcare-seeking behavior among cervical cancer patients and access to treatment facilities. Thus, we relied on estimates from the literature and varied parameters via sensitivity analysis. Fourth, HPV vaccine is targeting a population that is not routinely served under the current national immunization schedule. Based on in-country consultation and available literature, we made assumptions for vaccine coverage and estimated incremental health system costs associated with introducing HPV vaccine. We relied on published data in the absence of local empiric data. The incremental health system cost per dose applied in the study is relatively high (US$4.59) and sensitivity analysis showed that this parameter is a driver of results. Should Afghanistan manage to deliver HPV vaccine at a lower system cost, the value for money of HPV vaccination would substantially increase. The costs of treatment of cervical cancer estimated in this study are higher than that of other LMICs presented in the published literature [46]. Non-medical costs comprised a large portion (28% to 31%) of total treatment costs by stage, primarily driven by assumed costs of traveling abroad to receive radiotherapy services. However, our estimates were close to those from other published studies. For instance, treatment costs of local and regional/distant cancer were reported to be US$4492 and US$5387, respectively, in El Salvador [47], which are close to our health care cost estimates. To account for uncertainties in our input data, we conduct sensitivity analyses. However, our results do not include uncertainty intervals and the sensitivity analyses provide useful but incomplete results under uncertainty. Finally, our results should be viewed as conservative as we do not account for herd protection and our analysis excludes reductions in other HPV-related cancers.

The use of GDP-based cost-effectiveness thresholds (CETs) as a benchmark for whether the health gains offered by an intervention are large enough to justify any additional costs as recommended by the Commission on Macroeconomics in Health has recently been challenged [48], [49]. WHO updated these recommendations calling for countries to establish their own specific willingness-to-pay thresholds, taking into consideration factors such as affordability and feasibility [50]. Woods et al. predicted CET values for a list of LMICs and, according to their study, for Afghanistan CET would range from US$19 to US$349 [51]. However, the local expert team in Afghanistan elected to use one times the GDP per capita ($586) as a threshold for cost-effectiveness and a proxy of the government′s willingness to pay, in line with the country′s current practice for such evaluation.

As there are no organized screening and treatment options available in the country, HPV vaccination should receive additional consideration as a way to reduce cervical cancer burden in the country. Investing in women′s health through prevention of cervical cancer and other diseases can have positive effects that extend beyond the health costs and consequences included in this analysis, such as downstream impact on mental health, education, and economic status of family members. These and other non-healthcare sector costs were not included in our analysis but warrant consideration when evaluating the potential impact of preventing HPV morbidity and mortality among women in Afghanistan [52].

## 13 Conclusion

In Afghanistan, introduction of HPV vaccine is likely to be cost-effective compared to no vaccine according to the local willingness-to-pay threshold of one times the GDP per capita of US$586. Incremental cost-effectiveness ratios for HPV vaccination with the bivalent vaccine are projected to be US$426 and US$200 per DALY averted without and with a catch-up campaign, respectively, from the government perspective, and US$400 and US$174 per DALY averted from the societal perspective. In all scenario explored, including a catch-up campaign with the introduction of routine HPV immunization was more cost-effective than routine immunization alone. In addition to reducing burden on the health system, preventing downstream disease would reduce the financial and economic shocks imposed on women and their families. Importantly, cost-effectiveness estimates need to be considered in tandem with vaccination program affordability and feasibility for the government.

## 14 Authors′ contribution

Palwasha Anwari and Frédéric Debellut contributed by conceptualizing and designing the study, acquired data, and conducted the analyses. They also were primarily responsible for drafting the article. Elisabeth Vodicka, Andrew Clark, Farhad Farewar, Zubiada A. Zhwak, Dastagger Nazary, Clint Pecenka, D. Scott LaMontagne, and Najibullah Safi assisted in acquiring data and analysis and drafting. All authors approved the final submitted work.

## 15 Source of funding

This work was supported by the Bill & Melinda Gates Foundation, Seattle, WA [grant number OPP1147721].

## Declaration of Competing Interest

The authors declare that they have no known competing financial interests or personal relationships that could have appeared to influence the work reported in this paper.
