# Supplementary material for: Potential health impact and cost-effectiveness of bivalent human papillomavirus (HPV) vaccination in Afghanistan
Source: Vaccine. 2020 Feb 5;38(6):1352–62. doi: 10.1016/j.vaccine.2019.12.013 (PMC6997884; doi:10.1016/j.vaccine.2019.12.013)
Supplement: Supplementary Data 1 [file mmc1.docx]

**Annex 1: Patient Flow for Cervical Cancer in Afghanistan**

These diagrams illustrates patient flow of treatment seeking activities for cervical cancer in Afghanistan to support calculation of patient-level non-medical costs.


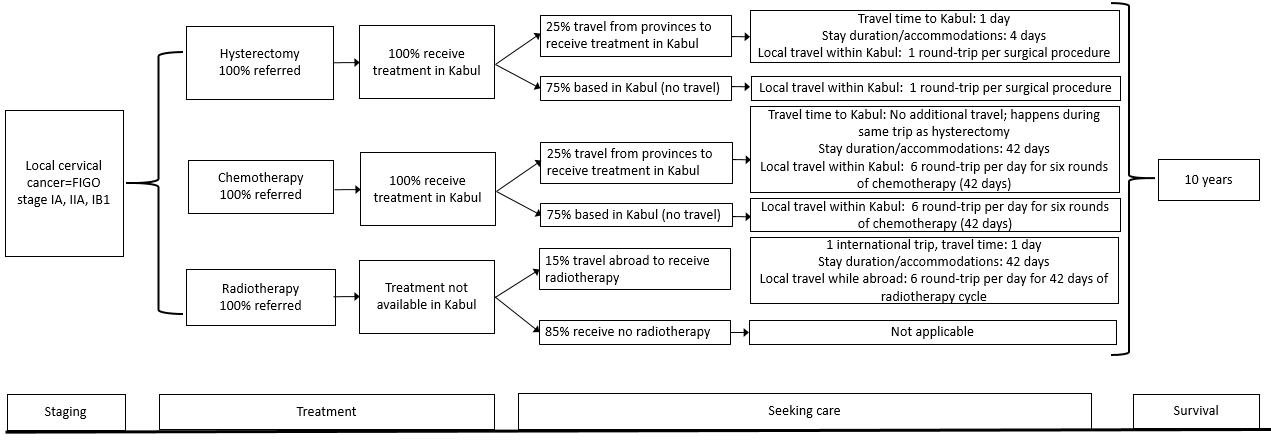


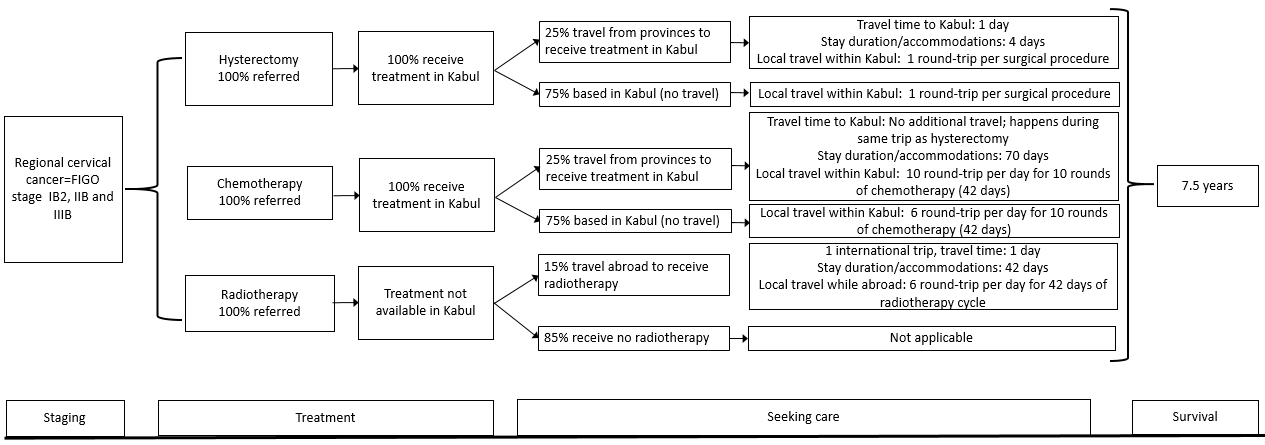


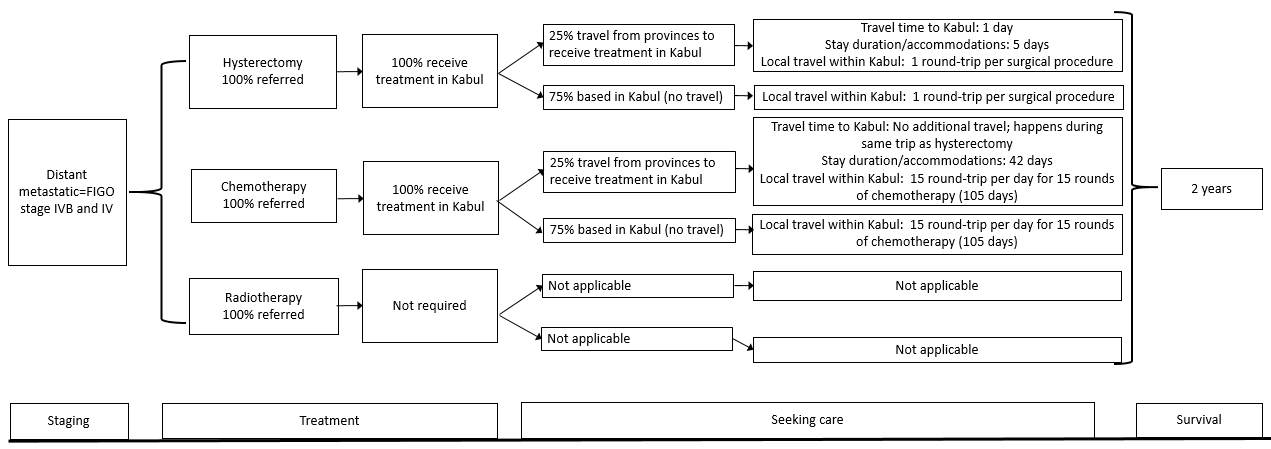


**Annex 2: Cervical cancer treatment cost calculation by cancer stage**

**Section 1: Calculation of direct medical costs**

We estimated direct medical costs of cervical cancer diagnosis and treatment of local, regional, and distant cervical cancer based on costs of care in private health facilities in Afghanistan. Unit cost data was collected from in-country experts including private hospital administrators and clinicians (Annex 2).

Cervical cancer treatment often requires a multifaceted and tailored approach based on individual characteristics, tumor size, and extension. To estimate an average cost of treatment for each type of invasive cancer, we applied the following definitions of staging and “standard treatment” based on guidelines set forth by the International Federation of Gynecology and Obstetrics (FIGO) ^[[1]](#footnote-1)^ and expert opinion from local oncologists.

1. Local cervical cancer corresponds to FIGO stage IA, IIA, and IB1. The standard treatment for IA and IIA for women with free margins consists of conization or simple hysterectomy. However, conization is not common practice in Afghanistan. In patients with positive margins, parametrial involvement or pelvic node involvement, standard treatment consists of hysterectomy plus chemo-radiation. There is no standard treatment for IB1, but options consist of surgery, external irradiation plus brachytherapy (radiotherapy) or combined radiosurgery. Our base case cost analysis assumed women with local cancer received treatment with hysterectomy. According to local oncologists, six rounds of chemotherapy plus radiation are also commonly prescribed to women with local invasive cancer in Afghanistan, Pakistan, and India. We assumed all women with local cancer would have hysterectomy.
2. Regional cervical cancer corresponds to FIGO stage IB2, IIB, IIIB. Our base case cost analysis assumed women with regional cancer received treatment with hysterectomy. According to local oncologists, 10 rounds of chemotherapy and radiation are commonly prescribed to women with regional invasive cancer in Afghanistan, Pakistan, and India.
3. Distant metastatic corresponds to FIGO stage IVB and IVA. Chemotherapy and palliative surgery are the main treatment regimen for this stage. Differences in chemotherapy schedules depends on pre-existing morbidity and potential toxicity for individualized treatment. Our base case analysis assumed women with distant cancer receive 15 rounds of chemotherapy, based on input from local oncologists regarding the number of chemotherapy rounds commonly prescribed for women with distant invasive cancer in Afghanistan, Pakistan, and India^[[2]](#footnote-2)^.

**Unit costs for base case cost calculations for treatment of local, regional and distant invasive cervical cancer**

In our cost calculation, we account for costs associated with diagnosis and treatment in private healthcare facilities. Table 1 presents all elements of costs in both 2018 Afghani (AFN-Afghanistan currency) and US$.

Table 1: Cost input parameters for health services in private facilities (2018 AFN and US$)

| **Categories** | **Cost per categories** | **Unit cost* in AFN** | **Unit cost in US$** |
| --- | --- | --- | --- |
| **Diagnosis** | Medical doctor visit (consulting fee) | 400 | 5.44 |
|  | Complete blood test | 1,500 | 20.41 |
|  | Histopathology test | 5,000 | 68.03 |
|  | Computed Tomography (CT) scan | 12,000 | 163.27 |
|  | Magnetic Resonance Imaging (MRI) | 13,000 | 176.87 |
| **Chemotherapy (per cycle)** | Chemotherapy medicine | 6,000 | 81.63 |
|  | Chemotherapy administration (includes medical doctor visit fee, nursing services, and bed occupancy for time of treatment) | 2,000 | 27.21 |
|  | Laboratory test required for each chemotherapy cycle | 1,500 | 20.41 |
| **Radiotherapy (per cycle)** | Average cost of a course of radiotherapy in India and Pakistan | 540,000 | 7,346.94 |
| **Relevant surgical procedures** | Hysterectomy | 35,000 | 476.19 |
|  | Surgical procedures for palliation (e.g., colostomy, bladder reconstruction, etc.) | 50,000 | 680.27 |
|  | Cost per bed night | 2,000 | 27.21 |
|  | Medicines during hospitalization such antibiotics, etc. | 5,000 | 68.03 |
|  | Laboratory test required for surgery | 1,500 | 20.41 |

*Unit costs for medical services reflect hospital fee charges to patients.

**Resource utilization assumptions for base case cost calculations for treatment of local, regional and distant invasive cervical cancer**

In Table 2, we present all services included in our base case for each stage of cervical cancer, including treatment components, frequency of each service used in treatment, price per service (i.e., unit price), and total cost of each treatment component. We assumed both local and regional cervical cancer would be treated with hysterectomy and chemotherapy and radiotherapy, when available, as per FIGO treatment guidelines and local common clinical practice in country. Hysterectomy is commonly recommended treatment for all local and regional cervical cancer cases.

Women were expected to receive six and ten rounds of chemotherapy for local and regional cancer, respectively. Radiotherapy is not currently available in Afghanistan. As such, women referred to radiotherapy must travel abroad (typically to India or Pakistan) to seek this treatment. In the base case, we assumed 15% of all cervical cancer patient would seek radiotherapy care abroad, based on estimates from local oncology experts. Women seeking radiotherapy abroad would still receive hysterectomy and chemotherapy in Afghanistan.

Palliative surgery, such as colostomy or reconstruction of bladder and chemotherapy, is the only treatment assumed for distant cancer. In our base case, we assumed all women would receive at least one palliative surgery (as tumor cells destroy normal pelvic anatomy), and 15 rounds of chemotherapy for distant cancer.

The unit prices were bundled fees for services by the hospital, so they already included staffing costs and facility-level non-medical costs, such as overhead cost. To avoid double counting, we did not account for staff costs separately.

| Table 2. Resource utilization assumptions for base-case cost calculations for treatment of local, regional, and distant invasive cervical cancer  All costs are in 2018 Afghani (AFN) ( US$ 1= 73.5 AFNs)^[[3]](#footnote-3)^ | | | | | | | | | | | | | |
| --- | --- | --- | --- | --- | --- | --- | --- | --- | --- | --- | --- | --- | --- |
| **Stage: Local** | | | | | | | | | | | | | |
|  | **Diagnosis** | | | | | **Medical treatment** | | | **Surgery treatment cost** | | | | **Radiotherapy** |
|  | Medical doctor visit | Complete blood test | Histopathology test | CT scan | MRI | Chemotherapy medicine | Chemotherapy administration* | Laboratory test required for each chemotherapy cycle | Surgery procedure** | Average duration of hospital stay (days) | Medicines provided during hospitalization, etc. | Laboratory test required for surgery | Average radiotherapy cost  (cost in India and Pakistan) |
| **Frequency/proportion** | 4 | 2 | 1 | 1 | 1 | 6 | 6 | 6 | 1 | 4 | 1 | 1 | 0.15 |
| **Unit price** | 400 | 1,500 | 5,000 | 12,000 | 13,000 | 6,000 | 2,000 | 1,500 | 35,000 | 2,000 | 5,000 | 1,500 | 540,000 |
| **Total cost in AFN** | 1,600 | 3,000 | 5,000 | 12,000 | 13,000 | 36,000 | 12,000 | 9,000 | 35,000 | 8,000 | 5,000 | 1,500 | 81,000 |
| **Total cost in US$** | 21.77 | 40.82 | 68.03 | 163.27 | 176.87 | 489.80 | 163.27 | 122.45 | 476.19 | 108.84 | 68.03 | 20.41 | 1,102.04 |
| **Stage: Regional** | | | | | | | | | | | | | |
| **Frequency/proportion** | 4 | 2 | 1 | 1 | 1 | 10 | 10 | 10 | 1 | 4 | 1 | 1 | 0.15 |
| **Unit price** | 400 | 1,500 | 5,000 | 12,000 | 13,000 | 6,000 | 2,000 | 1,500 | 35,000 | 2,000 | 5,000 | 1,500 | 540,000 |
| **Total cost in AFN** | 1,600 | 3,000 | 5,000 | 12,000 | 13,000 | 60,000 | 20,000 | 15,000 | 35,000 | 8,000 | 5,000 | 1,500 | 81,000 |
| **Total cost in US$** | 21.77 | 40.82 | 68.03 | 163.27 | 176.87 | 816.33 | 272.11 | 204.08 | 476.19 | 108.84 | 68.03 | 20.41 | 1,102.04 |
| **Stage: Distant** | | | | | | | | | | | | | |
| **Frequency/proportion** | 4 | 2 | 1 | 1 | 1 | 15 | 15 | 15 | 1 | 5 | 1 | 1 | NA |
| **Unit price** | 400 | 1,500 | 5,000 | 12,000 | 13,000 | 6,000 | 2,000 | 1,500 | 50,000 | 2,000 | 5,000 | 1,500 | 0 |
| **Total cost in AFN** | 1,600 | 3,000 | 5,000 | 12,000 | 13,000 | 90,000 | 30,000 | 22,500 | 50,000 | 10,000 | 5,000 | 1,500 | 0 |
| **Total cost in US$** | 21.77 | 40.82 | 68.03 | 163.27 | 176.87 | 1,224.49 | 408.16 | 306.12 | 680.27 | 136.05 | 68.03 | 20.41 | 0 |

*Chemotherapy administration includes medical doctor visit, nursing, and bed occupancy for time patients is receiving medicine
**Surgery procedure represents hysterectomy for local and regional cancer and palliative surgery (e.g., colostomy, bladder reconstruction, etc.) for distant cancer.

**Section 2: Calculation of patient-level direct non-medical costs**

We estimated direct non-medical costs to patients associated with diagnosis and treatment accounting for accommodation, food, and transportation costs incurred while seeking care. Since all female patients are accompanied by their male relative (called Mahram, referred to here as “attendee”), all direct non-medical costs accounted for two persons (patient and her attendee). In consensus with local experts’ opinion, we assumed that 25% of patients would travel from different provinces to Kabul to receive medical care; for these patients, we applied the average cost of round-trip bus fare for travel to Kabul. Within Kabul, we anticipated additional transport needs for every patient and her attendee to travel to and from the health facility. We assumed that the 25% of patients traveling to Kabul from a different province would require accommodations for the duration of their medical care; thus, we applied an estimated accommodation cost of AFN1,000 for those patients based on local experts’ estimation. Finally, we assumed that 15% of patients would receive treatment with hysterectomy and chemotherapy in Afghanistan but travel internationally to India and Pakistan for radiotherapy. For these women, we applied an average cost of travel to these two countries, according to private hospital administers and expert consultation. The average cost for travelling to India and Pakistan is inclusive of international flights, local transport in those countries, accommodation, and food for both the patient and her attendee.

Table 3: Unit costs of patient-level non-medical resources

| **Cost categories** | Interstate travel (round-trip) | Local transport  (round-trip) | Accommodation  (cost per night) | Meal  (cost per meal) | Inclusive average international travel to India and Pakistan* |
| --- | --- | --- | --- | --- | --- |
| **Unit price in AFN** | 6,000 | 1,000 | 1,000 | 250 | 66,150 |
| **Unit price in US$** | 81.63 | 13.61 | 13.61 | 3.4 | 900 |
| **Proportion of patients we account for these cost categories** | 0.25 | 1 | 0.25 | 1 | 0.15 |

*Travel to India and Pakistan is inclusive of international flights, local transport in those countries, accommodation and food for both the patient and her attendee.

Table 4: Non-medical resource utilization assumptions per patient by cancer stage

| **Cost categories*** | **Travel from provinces to Kabul , if relevant (# round trips)** | **Local transport to/from health facility**  **(# round-trips)** | **Accommodation for women traveling from outside Kabul (# nights)** | **# Meals required per person**  **(# meals) (total number of meals X 3 times in a day)** | **Average international travel to India and Pakistan, if relevant  (# round trips)** | **Remarks** |
| --- | --- | --- | --- | --- | --- | --- |
| **LOCAL** | **1** | **20** | **102** | **312** | **1** |  |
| **Diagnosis (including 4 gynecologist visits, blood tests, histopathology, CT scan and MRI)** | 1 | 7 | 14 | 45 |  | It generally takes two weeks to get results of laboratory testing and complete all diagnostic requirements. |
| **Chemotherapy** | Completed during same trip as diagnosis | 6 | 42 | 126 |  | Each chemo cycle is given one week apart. For 6 cycles, patients come to hospital/center to receive her treatment every week for six weeks. We assumed that patients traveling to Kabul from another province stayed in Kabul for the entire 6 weeks. |
| **Surgery (hysterectomy)** | Completed during same trip as diagnosis | 1 | 4 | 12 |  | On average, a patient stays for 4 nights in the hospital for Hysterectomy. Therefore, we accounted accommodation for her attendee and food for one person. |
| **Radiotherapy** | Not available in Kabul | 6 | 42 | 129 | 1 | Radiotherapy cycle is usually completed in 6 weeks (42 days). Radiotherapy is provided before chemotherapy. We assumed that women traveling abroad for radiotherapy stayed for the entire 6 week cycle. |
| **REGIONAL** | **1** | **24** | **130** | **396** | **1** |  |
| **Diagnosis (including 4 gynecologist visits, completing blood tests, histopathology, CT scan and MRI)** | 1 | 7 | 14 | 45 |  | It generally takes two weeks to get results of laboratory testing and complete all diagnostic requirements. |
| **Chemotherapy** | Completed during same trip as diagnosis | 10 | 70 | 210 |  | Each chemo cycle is given a week apart. For 10 cycles, patients come to hospital/center to receive her treatment every week for 10 weeks. |
| **Surgery (hysterectomy)** | Completed during same trip as diagnosis | 1 | 4 | 12 |  | On average, a patient stays for 4 nights in the hospital for Hysterectomy. Therefore, we accounted accommodation for her attendee and food for one person. |
| **Radiotherapy** | Not available in Kabul | 6 | 42 | 129 | 1 | Radiotherapy cycle is usually completed in 6 weeks (42 days). Radiotherapy is provided before chemotherapy |
| **DISTANT** | **1** | **23** | **124** | **375** | NA |  |
| **Diagnosis (including 4 gynecologist visits, completing blood tests, histopathology, CT scan and MRI)** | 1 | 7 | 14 | 45 |  | It takes two weeks to get results of laboratory testing and complete all diagnostic requirements. |
| **Chemotherapy** | Completed during same trip as diagnosis | 15 | 105 | 315 |  | Each chemo cycle is given a week apart. For 15 cycles, patients come to hospital/center to receive her treatment every week for 15 weeks. During this phase of treatment, patients coming from provinces need accommodations and meals. |
| **Surgery (palliation)** | Completed during same trip as diagnosis | 1 | 5 | 15 |  | On average, a patient stays for 5 nights in the hospital for palliative surgery. Therefore, we accounted for accommodation for her attendee and food for one person. |
| **Radiotherapy** | NA | NA | NA | NA | NA |  |

*Note: All costs reflect per-person costs for women seeking each type of treatment.

Table 5: Total non-medical costs per resource and resource assumptions by stage

All costs are in 2018 Afghani (AFN (US$ 1= 73.5 AFNs)

| **Local** | | | | | | |
| --- | --- | --- | --- | --- | --- | --- |
|  | **Travel from provinces to Kabul** | **Local transport** | **Accommodation** | **Food** | **Average international travel to India and Pakistan** | **Total** |
| **Frequency/ number days** | 1 | 22 | 102 | 312 | 1 |  |
| **Proportion** | 0.25 | 1 | 0.25 | 0.25 | 0.15 |  |
| **Unit cost in AFN in average** | 6,000 | 1,000 | 1,000 | 250 | 66,150 |  |
| **Total in AFN, per person** | 1,500 | 22,000 | 25,500 | 19,500 | 9,922.5 | 78,422.5 |
| **Total in AFN, for woman and male attendee** | 3,000 | 22,000 | 25,500 | 39,000 | 19,845 | 109,345.0 |
| **Total in US$** | 40.82 | 299.32 | 346.94 | 530.61 | 270 | 1,487.69 |
| **Regional** | | | | | | |
| **Frequency/ number days** | 1 | 26 | 130 | 396 | 1 |  |
| **Proportion** | 0.25 | 1 | 0.25 | 0.25 | 0.15 |  |
| **Unit cost in AFN in average** | 6,000 | 1,000 | 1,000 | 250 | 66,150 |  |
| **Total in AFN, per person** | 1,500 | 26,000 | 32,500 | 24,750 | 9,922.5 | 94,672.50 |
| **Total in AFN, for woman and male attendee** | 3,000 | 26,000 | 32,500 | 49,500 | 19,845 | 130,845.00 |
| **Total in US$** | 40.82 | 353.74 | 442.18 | 673.47 | 270 | 1,780.20 |
| **Distant** | | | | | | |
| **Frequency/ number days** | 1 | 25 | 124 | 375 | NA |  |
| **Proportion** | 0.25 | 1 | 0.25 | 0.25 |  |  |
| **Unit cost in AFN in average** | 6,000 | 1,000 | 1,000 | 250 |  |  |
| **Total in AFN** | 1,500 | 25,000 | 31,000 | 23,437.5 |  | 80,937.50 |
| **Total in AFN, for woman and male attendee** | 3,000 | 25,000 | 31,000 | 46,875 |  | 105,875.00 |
| **Total in US$** | 40.82 | 340.14 | 421.77 | 637.76 |  | 1,440.48 |

**Section 3: Calculation of indirect costs**

To assess indirect costs of treatment, we calculated the opportunity costs of seeking care for both the female patient and her attendee. For each treatment strategy, we estimated the number of days the pair would need to miss from work or usual activities due to diagnosis and treatment. For diagnosis and treatment, we assumed the following number of days would be required inclusive of medical visits and transportation: two day for round-road-trip to/from provinces to Kabul, 14 days for diagnosis, four days for surgery, 42, 70, and 105 days for chemotherapy for each local, regional, and distant case, and 40 days for radiotherapy plus two days for international travel (refer to Annex tables 4 and 6).

We calculated costs associated with loss of productivity based on annual per-capita Gross Domestic Product (GDP) of Afghanistan (US$586 in 2017)^[[4]](#footnote-4)^ per day (US$1.61) multiplied by the number of days lost. We estimated 104, 132, and 126 lost working days over the full course of diagnosis and treatment for local, regional, and distant cases respectively.

We calculated the total opportunity cost associated with cancer treatment, regardless of employment status, to take into consideration opportunity costs associated with non-monetized activities such as taking care of children and leisure activities. Even after remission, cancer care requires intensive follow-up. Treatment protocol requires a clinical visit with gynecological examination including Papanicolaou test (Pap smear) every three months for the first two years, every six months for the next three years, and yearly thereafter. Based on expert consensus, we assumed a patient with local cervical cancer would live for 10 years and therefore lose 19 working days for follow up visits, assuming each visit took a full day. We assumed a patient with regional cervical cancer would live for 7.5 years, therefore losing the equivalent of 17 working days for follow-up visits. We assumed a patient with distant cervical cancer would live for two years, hence losing about eight working days for follow-up visits.

Table 6: Total opportunity cost associated with cancer treatment by cancer stages:

| **Local** | | | | | | | | |
| --- | --- | --- | --- | --- | --- | --- | --- | --- |
| **Items** | **Travel from provinces to /from Kabul if relevant** | **Diagnosis** | | **Surgery** | **Chemotherapy** | **Radiotherapy** | **International round trip if applicable** | **Total** |
| **Number of days lost productivity** | 2 | 14 | | 4 | 42 | 40 | 2 | 104 |
| **Per-capita GDP per day in US$** | 1.61 | 1.61 | | 1.61 | 1.61 | 1.61 | 1.61 |  |
| **Full opportunity cost for patient (total # of days lost*GDP/day)** | 3.21 | 22.48 | | 6.42 | 67.43 | 64.22 | 3.21 | 166.97 |
| **Full opportunity cost for patient and her attendee** | 6.42 | 44.95 | | 12.84 | 134.86 | 128.44 | 6.42 | 333.94 |
| **Regional** | | | | | | | | |
| **Number of days lost productivity** | 2 | 14 | | 4 | 70 | 40 | 2 | 132 |
| **Per-capita GDP per day in US$** | 1.61 | 1.61 | | 1.61 | 1.61 | 1.61 | 1.61 | 1.61 |
| **Full opportunity cost for patient (total # of days lost*GDP/day)** | 3.22 | 22.48 | | 6.42 | 112.38 | 64.22 | 3.21 | 211.92 |
| **Full opportunity cost for patient and her attendee** | 6.44 | 44.95 | | 12.84 | 224.77 | 128.44 | 6.42 | 423.85 |
| **Distant** | | | | | | | | |
| **Number of days lost productivity** | 2 | 14 | 5 | | 105 | NA | NA | 126 |
| **Per-capita GDP per day in US$** | 1.61 | 1.61 | 1.61 | | 1.61 |  |  |  |
| **Full opportunity cost for patient (total # of days lost*GDP/day)** | 3.21 | 22.48 | 8.03 | | 168.58 | NA | NA | 202.29 |
| **Full opportunity cost for patient and her attendee** | 6.42 | 44.95 | 16.05 | | 337.15 | NA | NA | 404.58 |

**Summary - UNIVAC data inputs for cervical cancer treatment costs (in US$) for base, low, and high assumptions**

| **Base case** | **Direct medical cost** | **Direct non-medical cost** | **Indirect cost** | **Total** |
| --- | --- | --- | --- | --- |
| **Local cancer** | $3,021.77 | $1,460.48 | $333.94 | $4,816.18 |
| **Regional cancer** | $3,538.78 | $ 1,752.99 | $423.85 | $5,715.62 |
| **Distant cancer** | $3,314.29 | $ 1,413.27 | $404.58 | $5,132.13 |

Table 7: UNIVAC data inputs for cervical cancer treatment cost (in US$) for base case

Table 8: UNIVAC data inputs for cervical cancer treatment cost (in US$) for low scenario, where we do not account for radiotherapy abroad and its associated medical, non-medical, and indirect costs

| **Low scenario** | **Direct medical cost** | **Direct non-medical cost** | **Indirect cost** | **Total** |
| --- | --- | --- | --- | --- |
| **Local cancer** | $1,919.73 | $1,190.48 | $205.50 | $3,315.71 |
| **Regional cancer** | $2,436.73 | $1,482.99 | $295.43 | $4,215.15 |
| **Distant cancer** | $3,314.29 | $1,413.27 | $404.58 | $5,132.13 |

Table 9: UNIVAC data inputs for cervical cancer treatment cost (in US$) for high scenario, where we account for follow-up and its associated medical, non-medical, and indirect costs

| **High scenario** | **Direct medical cost** | **Direct non-medical cost** | **Indirect cost** | **Total** |
| --- | --- | --- | --- | --- |
| **Local cancer** | $3,125.17 | $2,752.99 | $394.95 | $6,273.11 |
| **Regional cancer** | $3,614.97 | $3,967.28 | $478.43 | $8,060.68 |
| **Distant cancer** | $3,357.82 | $2,828.23 | $430.27 | $6,616.32 |

1. Cervical cancer: ESMO clinical practice guidelines for diagnosis, treatment and follow up, C. Haie-Meder, P. Morice M. Castiglione, On behalf of the ESMO Guidelines Working Group; Annals of Oncology 21 (Supplement 5): v37–v40, 2010, doi:10.1093/annonc/mdq162 [↑](#footnote-ref-1)
2. Nicole G. Campos, Monisha Sharma, Andrew Clark, Kyueue Lee. et.al. The health and economic impact of scaling cervical cancer prevention in 50 low-and lower-middle-income countries, Int. Journal of Gynecology and Obstetrics, Supplement article, <https://doi.org/10.1002/ijgo.12184>, [Accessed Jan 26, 2019]  [↑](#footnote-ref-2)
3. Afghanistan Central Bank, Exchange rate dated July 1, 2018, <http://dab.gov.af/en/DAB/currency> [accessed 25 November 2018] [↑](#footnote-ref-3)
4. The World Bank , GDP per capita (current US$) <https://data.worldbank.org/indicator/NY.GDP.PCAP.CD?end=2017&locations=CN-AF&name_desc=true&start=1960&view=chart> [accessed 04 September 2018] [↑](#footnote-ref-4)
